# The psychological dimensions of sports injury risk: models, mechanisms, and interventions

**DOI:** 10.3389/fspor.2025.1690064

**Published:** 2025-11-21

**Authors:** Urban Johnson, Andreas Ivarsson

**Affiliations:** School of Health and Welfare, Halmstad University, Halmstad, Sweden

**Keywords:** biopsychosocial model, mindfulness interventions, psychological risk factors, sports injuries, stress response

## Abstract

Psychological factors influencing sports injury risk have received growing attention due to their potential for both prevention and rehabilitation. This mini review summarizes research related to psychological factors influencing sports injuries and strategies for their prevention. Injuries can have serious negative consequences for athletes' health and careers, as well as performance and financial implications for stakeholders. The research highlights psychological risk factors such as stress, anxiety, strong athletic identity, and pressure from coaches and teammates. The magnitude of stress responses is particularly linked to the risk of acute injuries, as outlined in Williams and Andersen's model and its evolution into the biopsychosocial framework. Recent studies have emphasized the complexity of overuse injuries, proposing multifactorial models accounting for intrapersonal, interpersonal, and sociocultural influences. Risk factors such as impaired neurocognitive functioning, academic stress, and strained coach-athlete relationships have been linked to both acute and overuse injuries. Combining psychological, physiological, and biomechanical assessments is increasingly recommended to better understand injury risk. Practical recommendations include fostering strong athlete-staff relationships, providing confidential psychological support, and integrating health education and autonomy-building into daily practice. Future research should investigate how combined psychological, physiological, and environmental factors affect injury risk. This mini review contributes to the evolving literature by highlighting how integrated psychological frameworks and intervention strategies can support evidence-based injury prevention and return-to-sport practices.

## Introduction

Experiencing a sports injury can lead to multiple consequences for both individuals and other stakeholders. At the individual level, injured athletes are at risk of various mental health issues, including elevated anxiety and depressive symptoms, as well as the potential involuntary termination of their athletic careers. For stakeholders, negative consequences such as decreased performance and financial losses may be associated with sports injuries [see e.g., (1)]. In recent years, an increasing number of scientific articles have emerged addressing psychosocial risk factors related to sports injuries, focusing on the prediction and prevention of both acute and overuse injuries. Consequently, **this mini review** summarizes recent developments in the psychological understanding and prevention of sports injuries, with a focus on biopsychosocial frameworks and intervention strategies. The objectives of this review are: (a) to summarize and present psychological risk factors for sports injuries, including leading theoretical frameworks; (b) to discuss research on psychological strategies aimed at reducing the risk of acute and overuse injuries, highlighting potential mechanisms for injury risk reduction; and (c) to provide recommendations for practical applications and further research.

## Methodology

To address the review objectives, we performed a narrative synthesis of peer-reviewed literature published between 2014 and 2024. The primary databases used for the search included PubMed and PsycINFO. Our focus was on high-quality sources such as systematic reviews, meta-analyses, and empirical studies that examined psychological risk factors, biopsychosocial frameworks, and injury prevention strategies. Studies were selected based on their relevance to psychological mechanisms and the etiology of both acute and overuse injuries in athletic populations. Key themes were identified through comparative analysis of theoretical models, cross-injury synthesis of findings, and assessment of reported intervention outcomes. This structured methodology ensures coherence between the review's aims and its conclusions, while also promoting transparency and reproducibility.

The review begins by briefly outlining the theoretical frameworks and highlighting primary risk factors identified in recent research as having the strongest associations with sports injury risk.

## Psychological risk factors and theoretical frameworks

### Risk factors for sports injuries: a psychological perspective

Several theoretical models have been developed in sports injury prevention and prediction research, primarily targeting acute injuries. One of the most cited frameworks is the model of stress and athletic injury (2). According to this model, an athlete's stress response, comprising attentional decrements (e.g., loss of sensitivity to peripheral cues and increased distractibility) and physiological changes (e.g., increased heart rate, muscular fatigue, and reduced neuromuscular control), to a potentially stressful event is hypothesized to have a direct effect on acute sport injury risk. Ivarsson et al. (3) conducted a systematic review with meta-analysis to further test this hypothesis and found that the stress response (e.g., lack of attention, concentration, and decreased processing speed) has the strongest relationship with acute sport injury risk compared with other components of the model, namely personality, history of stressors, and coping [see e.g., (4)].

Following the publication of the biopsychosocial model of stress, athletic injury, and health [BMSAIH; (5)], researchers expanded their investigative scope. The BMSAIH, built upon the revised model of stress and athletic injury, provides an understanding of mediating physiological mechanisms (e.g., stress hormone perturbation), other health conditions (e.g., illness), and behavioral influences (e.g., poor sleep) on sport injury risk. For instance, several general well-being factors have been associated with injury risk, including symptoms of depression (6), emotional exhaustion, fatigue, and decreased fitness or physical stress (7). These identified risk factors may also mediate the impact of stressors on injury risk. Other biopsychosocial factors tentatively increasing injury risk include sex, social load, and postpartum-related factors (8).

A multifactorial model addressing the etiology of overuse injuries is the overtraining risks and outcomes model (9). Within this model, interactions among psychosocial, intrapersonal, interpersonal, and situational factors are suggested to influence the risk of imbalance between stress and recovery. Based on the complex model for sports injury (10), Martin (11) proposed a complex psychosocial risk profile specifically for overuse injuries, highlighting recursive loops through which multiple iterations may occur between psychosocial determinants and overuse injury manifestations.

### Psychological risk factors for acute and overuse injuries

Sports injuries primarily result from a combination of factors; therefore, a complex sports approach is warranted [e.g., (10, 12)]. Within this context, various psychological risk and health factors have been proposed to increase the likelihood of sustaining a sports-related injury (13). As previously emphasized, history of stressors and stress responses are psychological variables most strongly associated with injury risk (3, 14).

More specifically, recent systematic reviews have shown that poorer neurocognitive function (i.e., slower visuomotor reaction time and processing speed) is associated with an increased risk of sport injuries [e.g., (15, 16)]. Moreover, various stressors, including academic stress (17), stressor-related disorders (18), and stress generated by teammates (19), may increase the risk of acute injury. Fewer studies have addressed overuse injuries. In a systematic review by Tranaeus et al. (20), several psychosocial factors were identified as potential risk factors for overuse injuries. Intrapersonal, interpersonal, and sociocultural factors were all identified as potential contributors. More specifically, in a study by Tranaeus et al. (20), intrapersonal factors (e.g., athletic identity), interpersonal factors (e.g., coach-athlete relationship), and sociocultural factors (e.g., pain normalization) were found to relate to overuse injury risk when synthesized through narrative analysis. These psychosocial factors and the potential mechanisms by which they may contribute to overuse injury development appeared to differ from those known for traumatic injuries. Athletes reporting the coach as a source of stress have also been found to have a greater risk of sustaining an overuse injury (19).

Another emerging research approach aligns with the biopsychosocial framework, combining risk factors from multiple disciplines in the same study. For example, Esmaeili et al. (21) found that a combination of risk factors, such as history of recent injuries, poorer mood, and reductions in musculoskeletal screening tests, was associated with an increased risk of injury. Similarly, Tranaeus et al. (20) showed that adequate coping strategies combined with high physical performance capacity were associated with reduced injury risk among female youth soccer players. These findings underscore the need for further research to adopt biopsychosocial approaches to enhance understanding of injury risk factors. While traditional stress-injury models focus primarily on acute injuries and individual psychological risk factors, current biopsychosocial and complex systems models emphasize multidimensional influences, including cultural and environmental factors. This shift marks a departure from linear causality to a more integrated understanding of injury etiology.

## Psychological strategies for injury prevention

### Intervention studies to prevent sports injuries

Research on psychological interventions aimed at reducing injury risk has been recognized for many years, both in junior (22) and senior athletes (23). The efficacy and effectiveness of various interventions have been demonstrated in several systematic reviews [e.g., (24)] and empirical studies in sport and exercise contexts [e.g., (25, 26)]. Most evidence-based research has focused on stress management programs (e.g., mindfulness, cognitive behavioral therapy), often delivered weekly in group settings [for summaries, see e.g., (14)].

There is also research focusing on cultural narratives (27) and associated resources such as “train smart, not hard” and developing a compassionate body-self relationship (28), which may further reduce injury risk. For example, a dominant cultural narrative in sport is the “culture of risk”, which downplays health and safety and encourages athletes to play through pain and injury (29). Considerable knowledge has highlighted cultural narratives and related resources that help reduce injury risk (30).

Psychological perspectives have also been integrated into studies on implementing traditional injury prevention training programs. Specifically, employing behavioral and social theoretical models, such as self-determination theory and the theory of planned behavior, during the implementation of exercise-based injury prevention programs, may increase adherence (31). Incorporating behavior change theories into delivery plans enhances the likelihood of sustainable behavioral change.

## Practical recommendations and future research

### Practical recommendations

Practical recommendations to prevent injury occurrence include fostering strong relationships among athletes and colleagues within the sporting environment (14), emphasizing shared goal consensus, collaboration, empathy, working alliance, and positive regard. Regular psychological support through confidential sessions could help athletes manage life challenges without stigma. Clubs should adopt a comprehensive approach addressing both individual and contextual risks (32). Improving health education and promoting player autonomy can enhance internal health control, raise pain awareness, encourage healthier behaviors, and mitigate fear of staff evaluation. Additionally, adopting mindfulness, acceptance-based practices, and stress management approaches to reduce acute injury risk may be beneficial, ideally combined with monitoring psychological factors such as psychosocial stress indices, sleep quality, and perceived recovery when screening for injury risk (33, 34).

It is also essential that coaches and support staff recognize the similarities and differences between traumatic and overuse injury etiologies (35) and their associated biopsychosocial markers. Furthermore, implementing injury prevention programs should incorporate behavior change theories to facilitate successful adoption within sports teams and organizations.

### Future research directions

Future research is recommended to investigate how combinations of risk factors across disciplines (e.g., psychology, physiology, biomechanics) influence injury risk. Additionally, merging research on environmental factors, available resources, and the needs and competencies of professionals working with athletes is essential when designing and evaluating novel psychological measures and interventions. One suggested intervention protocol is storytelling (36). Particularly for younger athletes, storytelling exercises aim to help them articulate their perspectives on others (e.g., friends, training partners, family). This transparent communication can foster self-awareness of their situations, feelings, and thoughts.

## Conclusion

In this review, we explored psychological risk factors and intervention strategies associated with both acute and overuse sports injuries, with a focus on developments in stress-injury and biopsychosocial models. Across the literature, psychological variables, particularly stress responses, coping capacity, and psychosocial stressors, consistently found to be key contributors to injury risk.

While stress management and mindfulness-based interventions show promise, questions remain regarding their long-term effectiveness and integration into multidisciplinary prevention strategies. The increasing shift toward biopsychosocial and complex systems frameworks marks meaningful progress by moving beyond linear explanations of injury. However, successful implementation will require greater methodological precision and collaboration across disciplines, particularly among sport psychologists, medical professionals, and coaches.
